# Bibliographical

**Published:** 1879-11

**Authors:** 


### BIBLIOGRAPHICAL.

### THE GUIDE TO SURGICAL DIAGNOSIS.

*By Christopher Heath, F. R. C. S., Holme Professor of Clinical Surgery, in University College, London.*

This work is just issued by Lindsay & Blakiston, of Philadelphia.

It is an interesting and valuable work to any one interested in, or connected with surgery.

In the introduction the author says, the object of the following work is to assist the student of surgery in forming a diagnosis of cases coming before hand.

Imperfect observation must lead to inaccurate or at least haphazzard diagnosis; and though experience may enable the surgeon to grasp the nature of the case as it were, instinctively, it would be most unsafe, for the inexperienced student to omit any of the steps by which an accurate diagnosis may be secured.

He most earnestly insists upon the practice of note taking in diagnosis, and that this should be done at the time of making the examination. He remarks that the briefest note made at the time of seeing a patient is infinitely more valuable, than an elaborate record penned hours or days afterwards.

How to observe, is an art to be obtained only by practice but what to observe in any given case, can be learned to a certain extent, from books.

This, it is the object of the following pages to furnish, so far as may be." Quite full details are given for case taking.

This is simply, and alone, a work on diagnosis, and no attempt is made to discuss the pathology or treatment of any of the diseases described.

The more salient points of the leading surgical affections of all the organs of the body are presented and clearly stated, in such a manner as to be readily comprehended by any one of ordinary surgical knowledge and skill.

This work is not only valuable to the general surgeon, but to those who practice specialties. In it the dental surgeon will find much valuable assistance. Chapter II. treats of the head, tumors and wounds of the scalp, fractures of the skull, concussion and compression of the brain. Chapter III. treats of affections of the face, the eyes, the ear, ulceration of face, affections of the nose. Chapter IV. of the mouth, the tongue, the teeth, the jaw—fracture and dislocation, of affections of the gums, tumors of the jaw, the palate. Chapter V. affections of the throat, acute and chronic dysphagia, dyspnœa, etc.

Thus it will be seen, that the entire region to which the special attention of the dentist is directed, is fully embraced.

The work will prove a valuable one in the hands of every dentist as well as surgeon.

